# Combining learning and constraints for genome-wide protein annotation

**DOI:** 10.1186/s12859-019-2875-5

**Published:** 2019-06-17

**Authors:** Stefano Teso, Luca Masera, Michelangelo Diligenti, Andrea Passerini

**Affiliations:** 1 Computer Science Department, KULeuven, Celestijnenlaan 200 A bus 2402, Leuven, 3001 Belgium; 2 Department of Information Engineering and Computer Science, University of Trento, Via Sommarive, 5, Povo di Trento, 38123 Italy; 3 Department of Information Engineering and Mathematics, University of Siena, San Niccolò, via Roma, 56, Siena, 53100 Italy

**Keywords:** Protein function prediction, Protein-protein interaction, Kernel methods, Genome annotation

## Abstract

**Background:**

The advent of high-throughput experimental techniques paved the way to genome-wide computational analysis and predictive annotation studies. When considering the joint annotation of a large set of related entities, like all proteins of a certain genome, many candidate annotations could be inconsistent, or very unlikely, given the existing knowledge. A sound predictive framework capable of accounting for this type of constraints in making predictions could substantially contribute to the quality of machine-generated annotations at a genomic scale.

**Results:**

We present Ocelot, a predictive pipeline which simultaneously addresses functional and interaction annotation of all proteins of a given genome. The system combines sequence-based predictors for functional and protein-protein interaction (PPI) prediction with a consistency layer enforcing (soft) constraints as fuzzy logic rules. The enforced rules represent the available prior knowledge about the classification task, including taxonomic constraints over each GO hierarchy (e.g. a protein labeled with a GO term should also be labeled with all ancestor terms) as well as rules combining interaction and function prediction. An extensive experimental evaluation on the Yeast genome shows that the integration of prior knowledge via rules substantially improves the quality of the predictions. The system largely outperforms GoFDR, the only high-ranking system at the last CAFA challenge with a readily available implementation, when GoFDR is given access to intra-genome information only (as Ocelot), and has comparable or better results (depending on the hierarchy and performance measure) when GoFDR is allowed to use information from other genomes. Our system also compares favorably to recent methods based on deep learning.

**Electronic supplementary material:**

The online version of this article (10.1186/s12859-019-2875-5) contains supplementary material, which is available to authorized users.

## Background

The advent of high-throughput experimental procedures comes both as an opportunity and as a challenge for computational approaches. On one hand, it allows to rely on unprecedented amounts of experimental data, such as sequential data at a genomic and meta-genomic scale as provided by NGS experiments. On the other hand, it calls for a change of scale for predictive approaches, from the focus on the analysis of individual biological sequences to the development of models characterizing the behavior of all sequences in a given genome or metagenome [1].

This level of analysis requires to develop models capable of *jointly* performing predictions on multiple entities, accounting for the relationships between these entities in order to provide predictions which are consistent with the existing knowledge.

In this paper we focus on two tightly connected aspects of protein behavior which are crucial in determining cell life, namely protein function and protein-protein interaction (PPI). By protein function we refer to the characterization of protein behavior as formalized by the Gene Ontology Consortium (GO) [2]. GO organizes the function of gene products into three hierarchies considering their molecular functions (MF), cellular compartments (CC) and biological processes (BP) respectively. Protein function prediction is one of the most popular bioinformatics tasks, as exemplified by the CAFA series [3] of protein function annotation assessments. Proteins mostly function through their interactions with other proteins, and predicting these interactions is thus at the heart of functional genomics [4]. Furthermore, PPI play crucial roles both in the mechanisms of disease [5] and the design of new drugs [6].

These predictive tasks are highly relational. GO hierarchies naturally enforce a set of taxonomic constraints between predictions. For instance, if a protein is annotated with a GO term it should also be annotated with the parents of this term (GO hierarchies are encoded as directed acyclic graphs) as well as with its ancestors, all the way up to the root of the hierarchy. Protein-protein interaction predictions provide additional sources of constraints, as for instance two interacting proteins are more likely to be involved in the same process, while two proteins located in different cellular compartments are less likely to interact.

Our predictive model is based on Semantic Based Regularization (SBR) [7], a statistical relational learning framework combining statistical learners with fuzzy-logic rules. For each GO term, a binary classifier is trained to predict whether a protein should be labeled with that term. A pairwise classifier is trained to predict whether pairs of proteins interact or not. All classifiers are implemented as kernel machines with kernels defined over multiple sources of information such as gene co-expression, sequence conservation profiles and protein domains (see Dataset construction for the details). Consistency among predictions is enforced by a set of fuzzy-logic rules relating terms in the hierarchies and terms with PPI predictions (see Methods for details).

An extensive experimental evaluation over the Yeast genome shows the potential of the approach. Yeast was chosen as a reference genome because of the large amount of functional and interaction annotation available. Our results show that both hierarchical and term-interaction rules contribute in increasing prediction quality in all GO hierarchies, especially for the lower levels where less training examples are available. PPI predictions provide an additional boost in function prediction performance. The converse is not true, as function predictions do not contribute to improve PPI prediction quality. This is an expected result, as the latter task is comparatively simpler, and information tends to propagate from simpler tasks to more complex ones. When compared to alternative approaches, our model substantially improves over GoFDR [8], the only high-ranking system at the latest CAFA challenge [3] for which an implementation was readily available, when GoFDR is allowed to access Yeast proteins only (as our method does), and has comparable or better results (depending on the hierarchy and performance measure) when GoFDR is given full access to the UNIREF90 database of proteins. In addition, our system produces comparable results to DeepGO [9], a deep learning-based method that relies on the true PPI network to produce its predictions.

The paper is structured as follows. In the next Section we position our contribution in the wider context of protein function prediction. We describe our prediction pipeline and constraints in “Methods” section, while “Results” section focuses on our experimental evaluation. We conclude with some final remarks in “Conclusion” section.

## Related work

Protein function prediction methods can be roughly grouped in two classes. Sequence-based methods perform annotation transfer by leveraging sequence similarity only. They follow a two-step scheme: first candidate homologues are identified using using tools like BLAST [10] or PSI-BLAST [11], then the annotations of the hits are transferred to the target based on various strategies. The underlying assumption is that homologues tend to share the same functions. Indeed, this is often the case for sequences with at least 60% similarity [12]. Targets that do not satisfy this condition are more challenging (they are referred to as “difficult targets” in CAFA parlance), and require finer-grained approaches. Recent approaches leverage deep learning architectures for analyzing the sequence data (e.g. [9]). Some sequence-based methods additionally rely on sequence features such as (inferred) domains, motifs, or conserved residues, see e.g. [8].

Data-based methods instead gather functional hints from heterogeneous data sources, including physical interactions [13, 14], co-expression patterns [15, 16], and genetic context [17, 18], among others. Please see [3, 19] for a list of frequently used sources. In this context, the key issue is how to appropriately integrate the sources while taking into account differences in format and reliability. The integration step is often carried out using statistical, probabilistic or machine learning tools.

Methods in both categories often do not enforce consistency among predictions. Those that do typically rely on a post-processing step to prune inconsistent annotations. More principled methods account for relations among GO terms directly in the training procedure, allowing annotation information to propagate across related terms. For instance, GOstruct [18, 20] employs structured output support vector machines (SVM) [21] to jointly predict all functional annotations of any target protein in a consistent manner. OCELOT follows the same principles, but relies on Semantic Based Regularization, a different, sound structured-output method. SBR has previously been applied to multi-level PPI prediction [22]. Contrary to structured-output SVMs, SBR can be easily adapted to different prediction tasks by changing the consistency rules, as described in Methods. Further, SBR does not require to solve an optimization problem explicitly (as is the case for loss-augmented inference in structured-output SVMs [21]) and can scale to larger tasks.

We note in passing that self-consistency alone is not enough to guarantee state-of-the-art results, as shown by the GOstruct results in the latest CAFA challenge [3]. More generally, despite the growing amount of “omics” data, which should favor data-based methods, sequence-based approaches proved to be hard to beat in practice [23], with some of them ranking among the top methods in the CAFA 2 competition [3]. For instance, GoFDR [8], an advanced sequence-based method, demonstrated excellent results in several categories, including eukaryotic genomes. Due to its excellent performance and immediate availability, we use GoFDR as the prime competitor in our experiments.

In addition, given the recent success of deep learning-based methods, we consider also the DeepGO approach of Kulmanov et al. [9]. This approach applies a one-dimensional convolutional neural network (with max-pooling layers) to the sequence data in order to produce a hidden representation of the protein. Then, PPI information is also converted into a hidden representation via knowledge graph embeddings. These representations are fed into a neural network, whose structure mimics the target GO ontology. DeepGO has shown considerable performance, but, in contrast to our method, it requires interaction data to be available.

## Methods

### Overview of the prediction pipeline

**Fig. 1:**
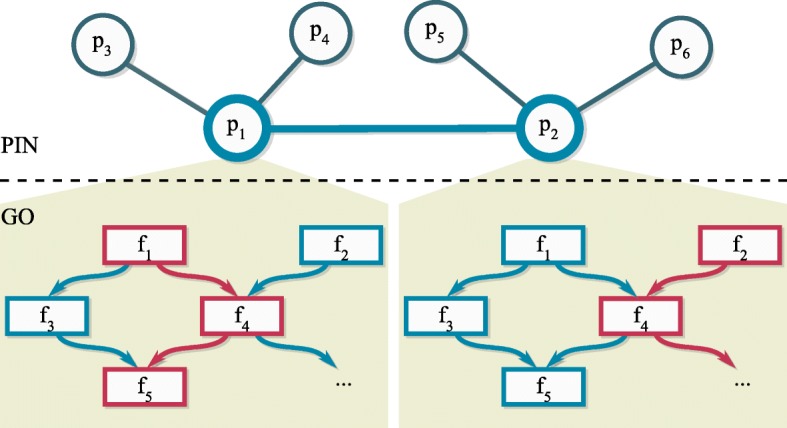
Depiction of the Ocelot decision making process. Above: predicted protein–protein interaction network, circles are proteins and lines represent physical interactions. Below: GO taxonomy, boxes are terms and arrows are IsA relations. Predicted annotations for proteins *p*_1_ and *p*_2_ (black): *p*_1_ is annotated with terms *f*_1_,*f*_4_,*f*_5_ and *p*_2_ with *f*_2_,*f*_4_. The functional predictions are driven by the similarity between *p*_1_ and *p*_2_, and by consistency with respect to the GO taxonomy (e.g. *f*_1_ entails either *f*_3_ or *f*_4_,*f*_2_ entails *f*_4_, etc.). The interaction predictions are driven by similarity between protein pairs (i.e. (*p*_1_,*p*_2_) against all other pairs) and are mutually constrained by the functional ones. For instance, since *p*_1_ and *p*_2_ do interact, OCELOT aims at predicting at least one shared term at each level of the GO, e.g. *f*_4_ at the middle level. These constraints are not hard, and can be violated if doing so provides a better joint prediction. As an example, *p*_1_ is annotated with *f*_1_ and *p*_2_ with *f*_2_. Please see the text for the details

Genome-wide prediction of protein function and interaction involves inferring the annotations of all proteins in a genome. OCELOT approaches this problem by decomposing it into simpler prediction tasks, and exploits prior biological knowledge to reconcile the resulting predictions. OCELOT instantiates one task for every candidate GO term, i.e., deciding whether a given protein should be annotated with that term, plus a separate task for deciding whether a given protein pair interacts. The overall, genome-wide annotations are obtained by imposing consistency across the predictions of all tasks. See Fig. 1 for a simplified depiction of our prediction pipeline.

In order to model the genome-wide prediction task, OCELOT employs Semantic Based Regularization (SBR) [7, 24], a state-of-the-art Statistical Relational Learning framework specifically designed to reason and learn with constraints and correlations among related prediction tasks. Entities, tasks and relations are encoded in SBR using First-Order Logic (FOL). At the logical level, proteins and terms are represented as constants *p,p*^′^,*f,f*^′^,*etc*, while annotations are modelled as predicates. OCELOT uses several predicates: a predicate *Fun*_*f*_(*p*) for each candidate term *f*, indicating whether protein *p* performs function *f*, and a separate predicate *Bound*(*p,p*^′^), encoding whether proteins *p* and *p*^′^ are physically bound. The truth value of a predicate is either fixed, in case the corresponding annotation is already known, or automatically imputed by SBR. In the latter case, the predicate is said to be a “target” predicate, and the truth value is predicted by a kernel machine [25, 26] associated to the predicate itself.

The kernel function, which lies at the core of kernel machines, measures the similarity between objects based on their representations. In our setting, a protein can be represented by the sequence of its residues, as well as by information about its amino acid composition or phylogenetic profile: having similar sequences, composition or profiles increases the similarity between proteins. Given a kernel and an object *x*, a kernel machine is a function that predicts some target property of *x* based on its similarity to other objects for which that property is known. More formally, the function is:

$$ \textstyle f(x) = \sum\nolimits_{i} w_{i} K(x, x_{i}) $$

This summation computes how strongly the property is believed to hold for *x* (if the sum is positive) or not (otherwise), and is often referred to as “confidence” or “margin”. For instance, a kernel machine could predict whether a protein *x* resides in the nucleus or not. In this case, being similar to a protein *x*_*i*_ residing in the nucleus (positive *w*_*i*_) drives the prediction toward a positive answer, while being similar to a protein *x*_*i*_ residing elsewhere (negative *w*_*i*_) has the opposite effect. Note that designing an appropriate kernel is critical for predictive performance.

In SBR each target predicate is implemented as a kernel machine. The truth value of a predicate—applied to an uncharacterized protein— is predicted by the associated kernel machine. Given a set of kernel machines (or predicates), SBR employs FOL rules to mutually constrain their predictions. It does so by first translating the FOL rules into continuous constraints using T-norms, a procedure discussed more thoroughly in “Semantic based regularization (SBR)” section. Roughly, these constraints combine the confidences (margins) of the predicates appearing in the FOL rule into an overall confidence in the satisfaction of the rule.

In order to make the predictions of different tasks consistent with the rules, SBR computes a joint truth value assignment that maximizes the sum of 1) the confidences of the individual predicates, and 2) the confidence in the satisfaction of the rules. Informally, the optimal assignment *y*^∗^ is obtained by solving the following optimization problem:

$$\begin{aligned} y^{*} = & \text{argmax}_{y} \; \text{consist}(y, \text{kernel machines}) \\&+ \text{consist}(y, \text{rules}) \end{aligned}$$

The two terms represent the consistency of the inferred truth values and with respect to the predictions given by the kernel machines, and with respect to the rules derived from the FOL background knowledge, respectively. Notice that in this optimization problem, the rules act as *soft* constraints, encouraging assignments satisfying many rules with high confidence.

As for most other complex Statistical-Relational Learning models [27], this inference problem is not convex, which implies that we are restricted to finding local optima. SBR exploits a clever two-stage procedure to improve the quality of the obtained local optimum. In a first step, SBR disables the constraints (by ignoring the second term of the equation above), thus obtaining individual predictions that fit the supervised data. This inference step is convex and can be solved efficiently to global optimality. In a second step, the obtained predictions are used as a starting point for the full inference procedure, where the constraints are turned back on. Empirically, this strategy was shown to achieve high-quality solutions, while being less computationally expensive than other non-convex optimization techniques [7].

SBR can be used both in inductive and transductive mode. In the latter case, both training and test examples are provided during training, with labels for the training examples only. In this way, test examples can contribute via the rule consistency term even if their labels are not known. Semi-supervised approaches are known to boost predictive performance [28], and fit the genome-wide prediction setting, where the full set of target proteins is available beforehand.

To summarize, functions and interactions of uncharacterized proteins are predicted based on similarity to other proteins and proteins pairs, respectively. The genome-wide predictions follow from applying consistency constraints, derived from biologically grounded FOL rules, to the low level predictions. In doing so, the constraints propagate information across GO terms and between the functional and interaction predictions.

### Rules

Functional annotations are naturally subject to constraints. We consider both constraints entailed by the Gene Ontology and constraints imposed by the (partially predicted) protein–protein interaction network. SBR allows to express these through First-Order Logic rules, and to efficiently reason over them, even in the presence of inconsistencies. We proceed to describe the rules employed by OCELOT.

**Consistency with the GO hierarchies.** The GO encompasses three domains, representing different aspects of protein function: biological process (BP), cellular component (CC), and molecular function (MF). Each domain specifies a term hierarchy, encoded as a directed acyclic graph: nodes are terms, while edges specify the specific-to-general isA relation^1^. More general terms (parents) are logically implied by more specific ones (their descendants). For instance, all proteins annotated with “ribosome” as their Cellular Component must also be annotated with its ancestor term “intracellular organelle”. We encourage the OCELOT predictions to be consistent with the GO with the two following constraints.

First, terms imply their parents. If a protein *p* is annotated with a term *f*, then it must also be annotated with all of its parent terms. The converse also holds: if *p* is not annotated with *f*, then it can not be annotated with any of its children either. These constraints can be expressed as a single FOL statement:

1 $$\begin{array}{*{20}l} & {Fun}_{f}(p) \Longrightarrow \bigwedge_{f^{\prime}\ \text{parent of}\ {f}} \; {Fun}_{f^{\prime}}(p) & \forall \, p \, \forall \, f \end{array} $$

Second, terms imply some of their children. If *p* is annotated with *f*, then it must be also annotated with at least one of the children of *f*:

2 $$\begin{array}{*{20}l} & {Fun}_{f}(p) \Longrightarrow \bigvee_{f^{\prime}\ \text{child of}\ {f}} \; {Fun}_{f^{\prime}}(p) & \forall \, p \, \forall \, f  \end{array} $$

Again, the converse also holds. These two rules are enforced for all GO aspects.

Note that if a protein is annotated (in the data) with a term *f* but with none of the children of *f*, the former may still result in the protein to be wrongly associated to a child term. We mitigate this applying the rules only to the upper levels of the hierarchy, where annotations are more abundant, as described below. Our empirical results show that, despite this issue, these rules provide non-negligible benefits in practice.

**Consistency with the interaction predictions.** Protein function and interactions are substantially intertwined: often a biological process is carried out through physical interaction, and interacting molecules must usually lie in the same (or close) cellular compartments. Functional annotations and interactions are tied together by requiring that binding proteins share at least one term at each depth of the corresponding domain. This defines one rule for each level of the considered GO hierarchy, which can be encoded in FOL as:

3 $$ Bound(p,p^{\prime}) \Longrightarrow \bigvee_{f \in \text{Domain}_{l}} \left({Fun}_{f}(p) \land {Fun}_{f}(p^{\prime}) \right) ~~~~~\!\forall \, p, p^{\prime}, l   $$

Here Domain_*l*_ is the set of GO terms appearing at depth *l* in the given domain. As above, the rule is soft. This rule is only applied to the BP and CC domains, as molecular function is less influenced by physical interactions. Further, we observed that this rule is mostly beneficial when applied to the top 5 levels of the CC taxonomy and 5 levels of the BP one. Its effect becomes irrelevant at the lower levels. Given that the rule is rather computationally expensive (as it involves all pairs of proteins *p,p*^′^ in the genome and all terms at each depth *l*), we opted for applying it to the upper levels only.

### Semantic based regularization (SBR)

**Knowledge Base and constraints.** SBR [7] is based on a variation of fuzzy generalizations of First Order Logic (FOL), which have been first proposed by Novak [29], and which can transform any FOL knowledge base into a set of real valued constraints.

A *T-norm fuzzy logic* [30] generalizes Boolean logic to variables assuming values in [0,1]. A T-norm fuzzy logic is defined by its T-norm *t*(*a*_1_,*a*_2_) that models the logical AND. A T-norm expression behaves as classical logic when the variables assume the crisp values 0 (false) or 1 (true). Different T-norm fuzzy logics have been proposed in the literature. For example, given two Boolean values $\bar {a}_{1}, \bar {a}_{2}$ and their continuous generalizations *a*_1_,*a*_2_ in [0,1], the Łukasiewicz T-norm is defined as $ (\bar {a}_{1} \land \bar {a}_{2}) ~~\rightarrow ~~ t(a_{1},a_{2}) = \max (0, a_{1} + a_{2} - 1) \ . $ The negation $\lnot \bar {a}$ of a variable corresponds to 1−*a* in the Łukasiewicz T-norm. From the definition of the ∧ and ¬ logic operators, it is possible to derive the generalized formulation for the ∨ operator via the DeMorgan law and the implication ⇒ via the T-norm residuum. Other choices of the T-norm are possible, like the *minimum T-norm* defined as $(\bar {a}_{1} \land \bar {a}_{2}) ~\rightarrow ~ t(a_{1},a_{2}) = \min (a_{1}, a_{2})$.

We focus our attention on FOL formulas in the Prenex Normal Form form, having all the quantifiers at the beginning of the expression. The quantifier-free part of the expression is an assertion in fuzzy propositional logic once all the quantified variables are grounded. Let’s consider a FOL formula with variables *x*_1_,*x*_2_,…, and let $\mathcal {P}$ indicate the vector of predicates and $\mathcal {P}(\mathcal {X})$ be the set of all grounded predicates.

The degree of truth of a formula containing an expression *E* with a universally quantified variable *x*_*i*_ is the average of the T-norm generalization *t*_*E*_(·), when grounding *x*_*i*_ over $\mathcal {X}_{i}$:

$$\begin{array}{l} \forall x_{i} ~~ E\left(\mathcal{P}(\mathcal{X})\right) ~~~\longrightarrow~ ~~ \Phi_{\forall}(\mathcal{P}\left(\mathcal{X})\right) = \frac{1}{|\mathcal{X}_{i}|} \sum\limits_{x_{i} \in \mathcal{X}_{i}} t_{E}\left(\mathcal{P}(\mathcal{X})\right) \end{array} $$

**Building constraints from logic.** Let us assume to be given a knowledge base *KB*, consisting of a set of FOL formulas. We assume that some of the predicates in the *KB* are unknown: the SBR learning process aims at finding a good approximation of each unknown predicate, so that the estimated predicates will satisfy the FOL formulas for the sample of the inputs. In particular, the function *f*_*j*_(·) will be learned by a Kernel Machine as an approximation of the *j*-th unknown predicate *p*_*j*_. Let ***f***={*f*_1_,…,*f*_*T*_} indicate the vector of all approximated predicates and $\boldsymbol {f}\left (\mathcal {X}\right)$ indicate the output values for all possible groundings of the approximated predicates. One constraint $1-\Phi _{i}(\boldsymbol {f}\left (\mathcal {X}\right))=0$ for each formula in the knowledge base is built by taking its fuzzy FOL generalization *Φ*_*i*_, where the unknown predicates are replaced by the learned functions.

**Cost function and training.** Let us assume that a set of *H* functional constraints 1−*Φ*_*h*_(***f***)=0,0≤*Φ*_*h*_(***f***)≤1, *h*=1,…,*H* describes how the functions should behave. Let $\boldsymbol {f}(\mathcal {X})$ be a vector collecting the values of the functions for each grounding. In order to enforce the functions to satisfy the constraints, the cost function penalizes their violation on the sample of data:

$$\begin{aligned} C_{e}[\boldsymbol{f}(\mathcal{X})] =& \sum\limits_{k=1}^{T} ||f_{k}||^{2} + \lambda_{l} \mathcal{L}(\boldsymbol{y}, \boldsymbol{f}(\mathcal{X}))\\ &+ \sum\limits_{h=1}^{H} \lambda_{h} \left(1 - \Phi_{h} \left(\boldsymbol{f}(\mathcal{X})\right) \right) \, \end{aligned} $$

where $\mathcal {L}(\boldsymbol {y}, \boldsymbol {f}(\mathcal {X}))$ is the loss with respect to the supervised examples ***y***,*λ*_*l*_ is the weight enforcing the fitting of the supervised patterns, *λ*_*h*_ is the weight for the *h*-th constraint and the first term is a regularization term penalizing non-smooth solutions such that ||*f*_*k*_||^2^=***w****kT****G***_*k*_***w***_*k*_, where ***G***_*k*_,***w***_*k*_ are the Gram matrix and the weight vector for the *k* function, respectively. The weights are optimized via gradient descent using a back-propagation schema, see [7] for more details.

**Collective classification.** The process of performing inference over a set of instances that are correlated is commonly referred to as *Collective classification* [31]. Collective classification takes advantage of the correlations by performing a collective assignment decision.

Let $\boldsymbol {f}(\mathcal {X}^{\prime })$ be a vector collecting the groundings for all functions over the test data. Collective classification for SBR minimizes the following cost function to find the values $\bar {\boldsymbol {f}}(\mathcal {X}^{\prime })$ respecting the FOL formulas on the test data:

$$\begin{aligned} C_{{coll}}\left[\bar{\boldsymbol{f}}(\mathcal{X}^{\prime}), \boldsymbol{f}(\mathcal{X}^{\prime})\right] =& \mathcal{L}_{{coll}} \left(\bar{\boldsymbol{f}}(\mathcal{X}^{\prime}), \boldsymbol{f}(\mathcal{X}^{\prime})\right) \\ &+ \sum\limits_{h} \left(1 - \Phi_{h}\left(\bar{\boldsymbol{f}} (\mathcal{X}^{\prime}) \right) \right) \ . \end{aligned} $$

where $\mathcal {L}_{{coll}}$ is a loss penalizing solutions that are not close to the prior values established by the trained kernel machines.

## Results

### Data processing

**Annotations** We built a comprehensive genome-wide yeast dataset. All data was retrieved in August 2014. Protein sequences were taken from the Saccharomyces Genome Database (SGD) [32]. Only validated ORFs at least 50 residues long were retained. The sequences were redundancy reduced with CD-HIT [33] using a 60% maximum sequence identity threshold, leading to a set of 4865 proteins. The identity threshold has been chosen in accordance with the *difficult* setting of the CAFA challenges [34].

Functional annotations were also taken from SGD, while the GO taxonomy was taken from the Gene Ontology Consortium website^2^. Following common practice, automatically assigned (IEA) annotations were discarded. We also removed all obsolete terms and mismatching annotations, i.e. SGD annotations that had no corresponding term in the GO graph. The resulting annotations were propagated up to the root, i.e. if a sequence was annotated with a certain term, it was annotated with all its ancestor terms in the hierarchy. Since known annotations become more sparse with term specificity, we discarded the lowest levels of each GO hierarchy: we retained terms down to depth 9 for Biological Process and Molecular Function, and down to 6 for Cellular Component. We also dropped terms that had fewer than 20 annotations^3^. Dropped annotations were ignored in our performance evaluation. The resulting dataset includes 9730 positive annotations. All missing annotations were taken to be negative^4^.

The protein–protein interaction network was taken from BioGRID [35]. Only manually curated physical interactions were kept. After adding any missing symmetric interactions, we obtained 34611 interacting protein pairs. An equal number of non-interactions was sampled from the complement of the positive protein–protein interaction network uniformly at random. This procedure is justified by the overwhelming proportion of true non-interactions in the complement [36]. All physical and functional interactions annotated in STRING 9.1 [37] were deleted from the complement prior to sampling, so to minimize the chance of sampling false negatives.

**Kernels** In OCELOT, each learned predicate is associated to a kernel function, which determines the similarity between two proteins (or protein pairs). Please see [25, 26] for background on kernel methods. Following the idea that different sources provide complementary information [18, 19, 38], we computed a number of kernels, focusing on a selection of relevant, heterogeneous biological sources, intended to be useful for predicting both functions and interactions. The sources include (i) gene *co-localization* and (ii) *co-expression*, (iii) protein *complexes*, (iv) protein *domains*, and (v) *conservation profiles*. Detailed explanations follow.

(i) Gene co-localization is known to influence the likelihood of proteins to physically interact [38], which is a strong indication of shared function [13, 14]. This information is captured by the gene co-localization kernel *K*_coloc_(*p,p*^′^)= exp(−*γ*|pos−pos^′^|). Here |pos−pos^′^| is the distance (measured in bases) separating the centroids of the genes encoding proteins *p* and *p*^′^. Closer centroids imply higher similarity. Genes located on different chromosomes have null similarity. Gene locations were obtained from SGD; *γ* was set to 1. (ii) Similarly, protein complexes offer (noisy and incomplete) evidence about protein–protein interactions [22, 38]. We incorporated this information through a diffusion kernel *K*_complex_(*p,p*^′^) over the catalogue of yeast protein complexes [39]. Roughly speaking, similarity between proteins is proportional to the number of shared binding partners (and their shared partners, and so on) the two proteins have. The exact values are defined in terms of a diffusion process over the complex network. The contribution of more distant partners is modulated by a smoothness parameter *β*, set to 1 in our experiments. We refer the reader to [40] for the mathematical details of diffusion kernels. (iii) Co-expression also provides valuable information [15]. The co-expression kernel is an inner product *K*_coexp_(*p,p*^′^)=〈***e***,***e***^′^〉 between vectors ***e*** and ***e***^′^ encoding the expression levels of *p* and *p*^′^ across experimental conditions. The measurements were taken from two comprehensive sets of micro-array experiments [41, 42] related to cell-cycle and environmental response in yeast. (iv) *Domains* often act as functional building blocks, so sharing the same domain is a strong indication of shared function [43]. We used InterPro [44] to infer the domains occurring in all proteins in the dataset. Presence of a domain in a protein *p* (resp. *p*^′^) is encoded by an indicator vector ***d*** (resp. ***d***^′^): the *k*-th entry of ***d*** is 1 if the *k*-th domain was detected as present in *p*, and zero otherwise. Given this information, we defined a linear kernel over the indicator vectors, i.e. $K_{\text {dom}}(p,p^{\prime }) = \sum \nolimits _{k} d_{k} d_{{k}^{\prime }}$. Similarity is determined by the number of shared domains. (v) Finally, we included phylogenetic information through a *profile* kernel [45, 46] over position-specific scoring matrices (PSSMs) obtained from the protein sequences. The PSSMs were computed with iterated PSI-BLAST (default parameters, two iterations) against the NCBI non-redundant sequence database (NR), as customary. Please see [45] for more details on profile kernels.

Each of the above kernels corresponds to a kernel 4865×4865 matrix. The matrices were normalized by the transformation $\hat {K}(p,{p^{\prime }}) = K(p,{p^{\prime }}) / \sqrt {K(p,p) \, K({p^{\prime }},{p^{\prime }})}$ and preconditioned by a small constant (10^−6^) for numerical stability. Since SBR allows only a single kernel for each target term, we aggregated all the matrices into a single one through simple averaging: $K(p,{p^{\prime }}) = \frac {1}{5} \sum \nolimits _{\text {all sources }}{s} \hat {K}_{s}(p,{p^{\prime }})$. This transformation equates to compounding information from all sources into a single kernel. More sophisticated strategies (e.g. assigning different weights to different kernels) did not provide any benefits in our empirical analysis. Finally, the interaction predicate works on *pairs* of proteins, and thus requires a kernel between protein *pairs*. Following Saccà et al. [22], we computed the pairwise kernel *K*_pairwise_((*p,p*^′^),(*q,q*^′^)) from the aggregate kernel *K*(*p,p*^′^) as follows:

$$\begin{aligned} K_{\text{pairwise}}((p,{p^{\prime}}),(q,{q^{\prime}})) =& K(p,q) \cdot K({p^{\prime}},{q^{\prime}}) \\&+ K(p,{q^{\prime}}) \cdot K({p^{\prime}},q) \end{aligned}$$

The pairwise kernel was also normalized and preconditioned.

### Empirical analysis

We assessed the performance of OCELOT by comparing it against several competitors: (i) GoFDR_U90_: the state-of-the-art GoFDR prediction method [8] trained over all sequences in UNIREF90 [47]. GoFDR is a state-of-the-art, sequence-based method that ranked very high in the CAFA 2 competition [3]. GoFDR^5^ was shown to perform well on both *difficult* and eukaryote targets. Note that UNIREF90 contains substantially more sequences than our own yeast genome dataset (including orthologues), giving GoFDR_U90_ a significant advantage in terms of sequence information. (ii) GoFDR_yeast_: GoFDR trained only on the same sequences used by OCELOT. Since only yeast sequences are considered, the parameters of PSI-BLAST (as used by GoFDR) were adjusted to capture even lower confidence alignments (namely by increasing the E-value threshold to 0.9 and the number of iterations from 3 to 4). (iii) BLAST: an annotation transfer approach based on BLAST, used as baseline in the CAFA2 competition^6^. (iv) OCELOT with only GO consistency rules (i.e. no protein–protein interactions), and with no rules at all. We refer to these two baselines as OCELOT_go_ and OCELOT_indep_, respectively.

All methods were evaluated in the *difficult* CAFA setting^7^ using a 10-fold cross-validation procedure: the proteins were split into 10 subsets, 9 of which were used for parameter estimation, and the remaining one for evaluation. The folds were constructed by distributing functional and interaction annotations among them in a balanced manner using a greedy procedure. Interactions were split similarly.

In addition, we also compared OCELOT against DeepGO [9], a state-of-the-art deep learning approach that exploits sequence and PPI data. In contrast to the other methods, the results for DeepGO were obtained from its web interface^8^. Having no control over the ontology used by DeepGO, we had to limit the comparison to the overall perfomance computed on the terms in common between our and DeepGO’s ontologies.

**Performance measures.** Following the CAFA2 procedure, predicted annotations were evaluated using both protein-centric and term-centric performance measures [3]. Protein-centric measures include the *F*_max_ and *S*_min_ scores, defined as:

$$\begin{aligned} {F_{\text{max}}} &= \max_{\tau \in [0, 1]} \frac{2 \, \text{pr}(\tau) \, \text{rc}(\tau)}{\text{pr}(\tau) + \text{rc}(\tau)} \\ {S_{\text{min}}} &= \min_{\tau \in [0, 1]} \sqrt{\text{ru}(\tau)^{2} - \text{mi}(\tau)^{2}} \end{aligned} $$

The *F*_max_ score is maximum value achieved by the *F*_1_ score, i.e. the harmonic mean of the precision pr(*τ*) and recall rc(*τ*):

$$\begin{aligned} \text{pr}(\tau) &= \frac{1}{m(\tau)} \sum\limits_{i=1}^{m(\tau)} \frac{|P_{i}(\tau) \cap T_{i}|}{|P_{i}(\tau)|} \\ \text{rc}(\tau) &= \frac{1}{n} \sum\limits_{i=1}^{n} \frac{|P_{i}(\tau) \cap T_{i}|}{|T_{i}|} \end{aligned} $$

Here *P*_*i*_(*τ*) is the set of predicted GO annotations for the *i*-th protein, *T*_*i*_ is the set of true (observed) annotations, *m*(*τ*) is the number of proteins with at least one predicted annotation at threshold *τ*, and *n* is the total number of proteins. The *S*_min_ score is the minimum semantic distance, defined in terms of the remaining uncertainty (ru) and misinformation (mi):

$$\begin{array}{*{20}l} \text{ru}(\tau) & = \frac{1}{n} \sum\limits_{i=1}^{n} \sum\limits_{f} \text{ic}(f) [\!\![ f \not\in P_{i}(\tau) \land f \in T_{i} ]\!\!] \\ \text{mi}(\tau) & = \frac{1}{n} \sum\limits_{i=1}^{n} \sum\limits_{f} \text{ic}(f) [\!\![ f \in P_{i}(\tau) \land f \not\in T_{i} ]\!\!] \end{array} $$

where ic(*f*) is the information content of term *f* and [ [·] ] is the 0-1 indicator function. Note that these metrics capture the overall quality of the learned model by explicitly optimizing the decision threshold *τ*. In order to capture the actual usage of the models, where the decision threshold can not be optimized directly, we also evaluated the predicted annotations using the *F*_1_ score, i.e. the *F*_max_ score with *τ* fixed to 0.5, as well as precision and recall with the same decision threshold *τ*=0.5. As in CAFA2, we used the Area under the Receiver Operating Characteristic Curve (AUC) for the term-centric evaluation.

**Fig. 2:**
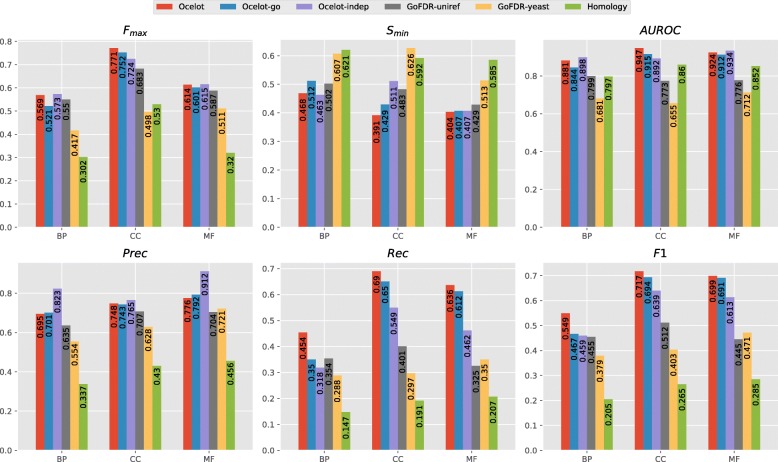
Overall performance of all prediction methods on the Yeast dataset. Best viewed in color

**Discussion** The overall performance of all predictors can be found in Fig. 2. At a high level, all prediction methods tend to perform better than both the simple BLAST baseline, as expected, and GoFDR_yeast_. This is hardly surprising: despite being configured to consider even distantly related homologues (by tweaking the PSI-BLAST parameters, as mentioned above), GoFDR_yeast_ could not transfer any annotations to 1133 targets, as no alignment could be found in the yeast-only training set. Allowing GoFDR to access extra-genomic sequences solves this issue, as shown by the improved performance of GoFDR_U90_ over GoFDR_yeast_.

On the other hand, OCELOT, OCELOT_go_, and OCELOT_indep_, perform as well or better than GoFDR_U90_ in terms of *F*_max_ and *S*_min_. The overall performance on BP and MF are rather close, while for CC the SBR-based methods offer a large improvement: the *F*_max_ and *S*_min_ of OCELOT are approximately 9% better (resp. higher and lower) than those of GoFDR_U90_.

More marked improvements can be observed in the *F*_1_ plots. The kernel-based methods perform as well or better than GoFDR_U90_ in all GO domains. This holds despite the task being very class unbalanced (especially at the lower levels of the hierarchy), and the decision threshold being fixed at 0.5. In CC and MF, the biggest contribution comes from the hierarchy consistency rules. In contrast, consistency to the protein–protein interaction network seems to be the biggest factor for BP: OCELOT offers an 8% *F*_1_ improvement over OCELOT_indep_,OCELOT_go_ and GoFDR_U90_.

**Fig. 3:**
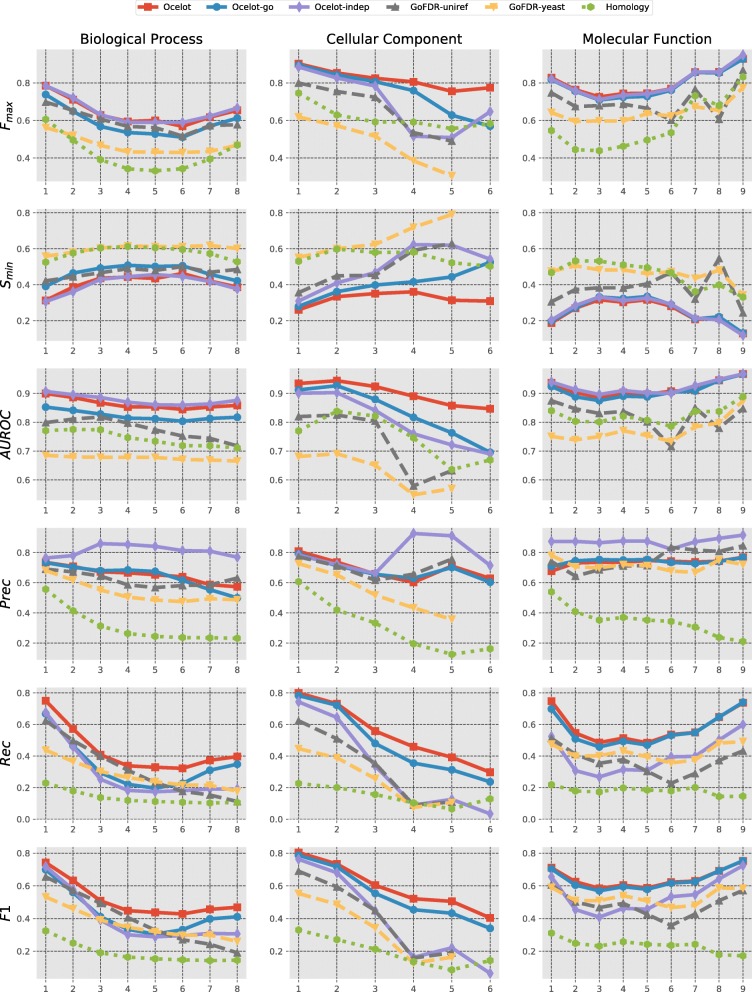
Breakdown of the performance of all methods at different GO term depth. Because GoFDR_yeast_ and GoFDR_U90_ predicted no labels for level 6 of cellular component, no metric is reported for the specific depth level. Best viewed in color

A breakdown of the performance at different term depths is provided in Fig. 3. The general trend is the same as above: all methods outperform the baseline and GoFDR_yeast_, and OCELOT with the full set of rules has the overall best performance. In all cases, the performance of the OCELOT_indep_ is comparable to that of OCELOT at the top levels, however it quickly degrades with term depth. This implies that the consistency rules are successfully propagating the correct predictions down the hierarchy. This is especially evident for the cellular component domain. For the molecular function domain, the bottom levels are predicted as good as the top ones, and much better than the intermediate levels. This is actually an artifact of the sparsity in annotations at the lowest levels (recall that we dropped terms with less than 20 annotations, which drastically reduces the number of terms which are predicted in the lowest levels, especially for MF).

Few examples can help highlighting the role of the rules to enforce consistency in predictions. For example, taxonomical consistency allows to recover some GO-terms for the MAS2 protein which are missed by OCELOT_indep_. The predictor correctly assigns the cytoplasmatic part GO-term to MAS2, but fails to identify its children terms mitochondrial-part and mitochondrion. OCELOT_go_ manages to recover these two terms thanks to the second taxonomical rule (Eq. 2). When also considering the consistency with respect to the PPI predictions, the protein-complex localization is also correctly predicted for the same protein.

Note that the boost in performance given by the PPI rules is achieved regardless of the fact that interactions are predicted and not observed. The PPI predictions performance are: 0.61 precision, 0.80 recall, 0.69 *F*_1_ and 0.72 AUC. These performance are only due to the kernels, and are not affected by the introduction of the GO rules^9^. As already mentioned, the fact that PPI prediction can not be significantly improved by exploiting their correlation with protein functions is an expected outcome. Indeed, PPI is comparatively a simpler prediction problem, and information tends to propagate from simpler to more complex tasks. A similar result has been observed in multi-level interaction prediction, where propagation flows from the protein to the domain and residue level but not viceversa [22].

**Fig. 4:**
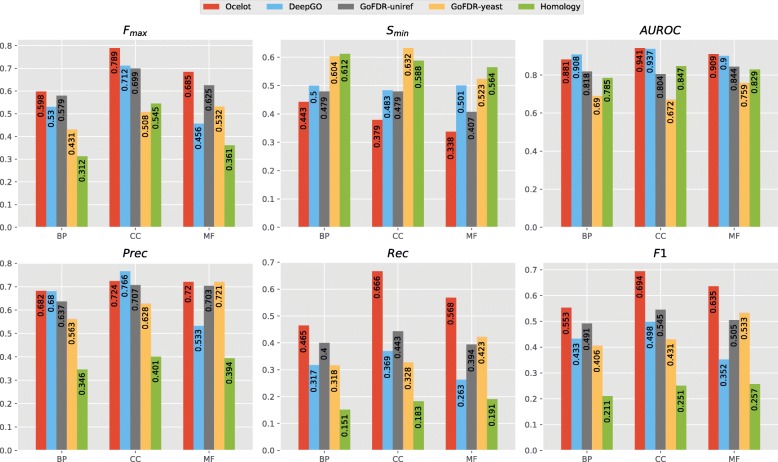
Overall performance of DeepGO, OCELOT, GoFDR and the baseline on the Yeast dataset. Best viewed in color

We also compared OCELOT to DeepGO, a state-of-the-art deep learning-based predictor [9]. Since we could not train DeepGO on our ontology, we compared the methods only on the terms shared by our and DeepGO’s ontology. The results are shown in Fig. 4. The results confirm the ones obtained by Kulmanov et al. [9], where DeepGO outperforms GoFDR in terms of AUC. On the other hand, OCELOT and DeepGO perform comparably, in terms of AUC and precision, with some slight variation between different aspects. Note that this holds regardless of the fact that DeepGO was trained on many more sequences than OCELOT, and that it uses true interaction data. In contrast, OCELOT has only access to yeast sequences, and only to predicted protein interactions. Most importantly, OCELOT outperforms DeepGO on all aspects for all other performance measures (*F*_max_,*S*_min_, recall and *F*_1_). The performance of DeepGO is especially poor under the *F*_1_ metric, showing that the predictor is not suitably calibrated against the natural decision threshold *τ*=0.5.

**Fig. 5:**
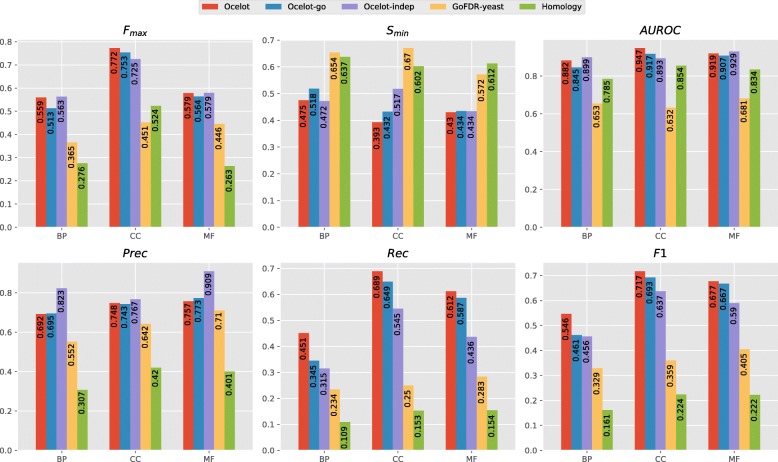
Overall performance of all prediction methods on the Yeast dataset filtered from remote homologies (sequence identity <25*%*). Best viewed in color

As a final experiment, we evaluated the performace of OCELOT and its competitors in a setting where not even remote homologies can be used to make predictions. We thus created a further reduced dataset by running psi-cd-hit [48] (as cd-hit does not support low sequence identity cutoffs) with a threshold at 25% sequence identity, in order to stay below the twilight zone of sequence alignment [49]. The resulting dataset is composed by 4140 proteins. The overall performance for the different methods is reported in Fig. 5. As expected, a general drop in performance can be observed with respect to the case with the threshold at 60% (see Fig. 2). It is however worth noticing that the drop is not the same among the tested methods. Indeed, Ocelot-based methods are just marginally affected by the harder setting, as they rely on multiple sources of information in addition to sequence similarity. On the other hand, both GoFDR_yeast_ and the baseline perform substantially worse, with a relative drop of more then 10% in *F*_max_ and 7% in *S*_min_. The breakdown of the performance, reported in Additional file 1, shows no significant difference in the performance trends with respect to the original setting.

## Conclusion

We introduced OCELOT, a predictive system capable of jointly predicting functional and protein-protein interaction annotations for all proteins of a given genome. The system combines kernel machine classifiers for binary and pairwise classification with a fuzzy logic layer enforcing consistency constraints along the GO hierarchy and between functional terms and interaction predictions. We evaluated the system on the Yeast genome, showing how the rule enforcement layer manages to substantially improve predictive performance in functional annotation, achieving results which are on par or better (depending on the GO domain and performance measure) than those of a state-of-the-art sequence-based approach fed with annotations from multiple genomes.

OCELOT can be extended in a number of directions. The system is currently conceived for intra-genome annotation. A first major extension consists of adapting it to process multiple genomes simultaneously. This requires to incorporate both novel specialized predictors, like an orthology-based annotator [50], and additional inter-genome rules, e.g. encouraging (predicted) orthologues to interact with the same partners. A second research direction consists in broadening the type of annotations provided by the system, by e.g. generalizing interaction prediction to the prediction of biochemical pathways [51]. Care must be taken in encoding appropriate rules in order to ensure consistent predictions without eccessively biasing the annotation.

## Availability and requirements

- Project name: Ocelot
- Project home page: https://sites.google.com/view/experimental-data/home
- Operating system(s): GNU/Linux, macOS
- Programming language: Python, C++
- License: BSD 3
- Any restrictions to use by non-academics: None

The datasets supporting the conclusions of this article are available in the Ocelot data repository, ftp://james.diism.unisi.it/pub/diligmic/OcelotData.

## Additional file

Additional file 1 Breakdown of the performance on the dataset filtered from remote homologies (sequence identity <25*%*) at different GO term depth. Because GoFDR_yeast_ predicted no labels for level 6 of cellular component, no metric is reported. Best viewed in color. (PDF 62 kb)

## Abbreviations

AUC: Area under the receiver operating characteristic curve
BP: Biological process
CC: Cellular compartment
FOL: First order logic
GO: Gene ontology
MF: Molecular function
PPI: Protein-protein interaction
SBR: Semantic based regularization
SGD: Saccharomyces genome database
SVM: Support vector machines

## Footnotes

1 In this paper we restrict ourselves to “isA” relationships only, since the remaining GO relations, e.g. “partOf” and “regulates”, occur too infrequently in the ontology.

2
http://geneontology.org/page/download-ontology

3 Annotations of dropped child terms were aggregated into new “bin” nodes under the same parent. These terms provide useful supervision during training, and increase the satisfaction of Ocelot rules; see below for details.

4 Some databases, e.g. NoGO [52], do publish curated negative functional annotations. However, these resources do not yet provide enough annotations for training our predictor. Therefore, we resorted to sampling negative annotations from the non-positive ones, as is typically done. We adopted the same solution for negative interaction annotations [53].

5 Software taken from http://gofdr.tianlab.cn.

6 Software taken from https://github.com/yuxjiang/CAFA2.

7 60% maximum sequence identity

8 The DeepGO package does not provide a procedure for training the model on our yeast dataset. The predictions were retrived from http://deepgo.bio2vec.net/deepgo/on 14th June 2018.

9 A main decision choice in using the SBR framework is the selection of the T-norm used to convert the rules in the knowledge-base into a differentiable constraint (see “Semantic based regularization (SBR)” section). While PPI prediction performance couldn’t be improved regardless of the choice of the T-norm, the largest improvements in function prediction were obtained when converting the logic rules defined in Eq. 3 using the minimum T-norm. The derivative of the residuum of the minimum T-norm with respect to the predicate outputs has the property of depending only on the value of the right side of the implication (e.g. the body of a clause). Therefore, this choice of T-norm makes the PPI predictions, corresponding to the output of the *Bound* predicate that appears only on the head of the rule, not affected by the function predictions. The converse is not true, and the function predictions are indeed significantly affected and improved by the PPI output values.
