# Supplementary figures and images for: Combining learning and constraints for genome-wide protein annotation
Source: BMC Bioinformatics. 2019 Jun 17;20:338. doi: 10.1186/s12859-019-2875-5 (PMC6580517; doi:10.1186/s12859-019-2875-5)

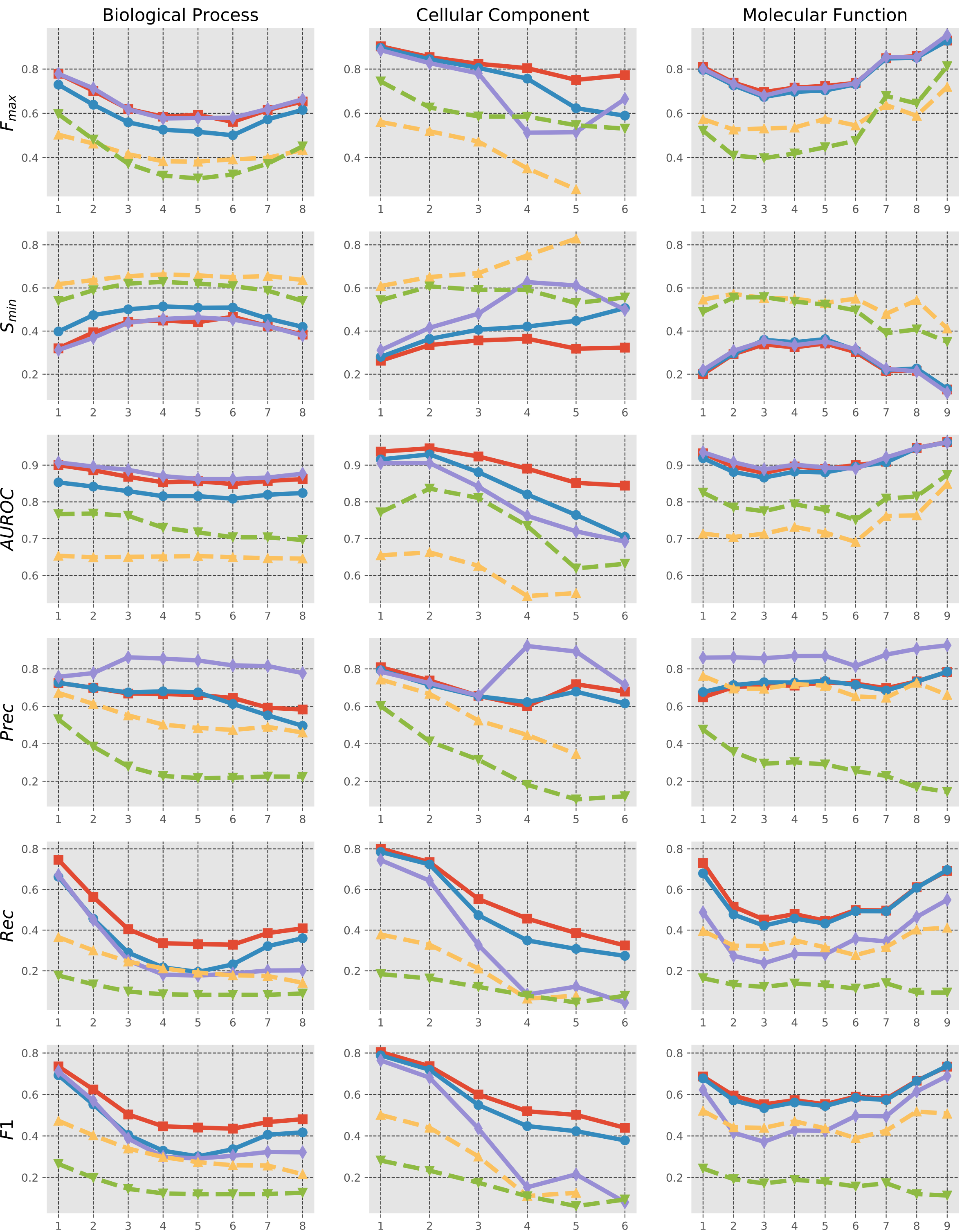

Supplement: Supplementary file 1 — Breakdown of the performance on the dataset filtered from remote homologies (sequence identity <25%) at different GO term depth. Because GoFDRyeast predicted no labels for level 6 of cellular component, no metric is reported. Best viewed in color. (PDF 62 kb) [file 12859_2019_2875_MOESM1_ESM.pdf]
